# Factors Predicting Older People’s Acceptance of a Personalized Health Care Service App and the Effect of Chronic Disease: Cross-Sectional Questionnaire Study

**DOI:** 10.2196/41429

**Published:** 2023-06-21

**Authors:** Jun Hyuk Koo, You Hyun Park, Dae Ryong Kang

**Affiliations:** 1 National Health BigData Clinical Research Institute Yonsei University Wonju Industry-Academic Cooperation Foundation Wonju Republic of Korea; 2 Department of Biostatics Yonsei University Graduate School Wonju Republic of Korea; 3 Department of Precision Medicine Yonsei University Wonju College of Medicine Wonju Republic of Korea

**Keywords:** environmental risk factor, personalized health care service app, chronic disease, unified theory of acceptance and use of technology, structural equation modeling, older adult, acceptance, adoption, technology use, mHealth, mobile health, mobile app, health app, gerontology, personalized, health care service, intention to use

## Abstract

**Background:**

Mobile health (mHealth) services enable real-time measurement of information on individuals’ biosignals and environmental risk factors; accordingly, research on health management using mHealth is being actively conducted.

**Objective:**

The study aims to identify the predictors of older people’s intention to use mHealth in South Korea and verify whether chronic disease moderates the effect of the identified predictors on behavioral intentions.

**Methods:**

A cross-sectional questionnaire study was conducted among 500 participants aged 60 to 75 years. The research hypotheses were tested using structural equation modeling, and indirect effects were verified through bootstrapping. Bootstrapping was performed 10,000 times, and the significance of the indirect effects was confirmed through the bias-corrected percentile method.

**Results:**

Of 477 participants, 278 (58.3%) had at least 1 chronic disease. Performance expectancy (β=.453; P=.003) and social influence (β=.693; P<.001) were significant predictors of behavioral intention. Bootstrapping results showed that facilitating conditions (β=.325; P=.006; 95% CI 0.115-0.759) were found to have a significant indirect effect on behavioral intention. Multigroup structural equation modeling testing the presence or absence of chronic disease revealed a significant difference in the path of device trust to performance expectancy (critical ratio=–2.165). Bootstrapping also confirmed that device trust (β=.122; P=.039; 95% CI 0.007-0.346) had a significant indirect effect on behavioral intention in people with chronic disease.

**Conclusions:**

This study, which explored the predictors of the intention to use mHealth through a web-based survey of older people, suggests similar results to those of other studies that applied the unified theory of acceptance and use of technology model to the acceptance of mHealth. Performance expectancy, social influence, and facilitating conditions were revealed as predictors of accepting mHealth. In addition, trust in a wearable device for measuring biosignals was investigated as an additional predictor in people with chronic disease. This suggests that different strategies are needed, depending on the characteristics of users.

## Introduction

### Background

New medical strategies that aim to change individual behaviors and lifestyles are being introduced. These medical strategies are based on information and communications technology and the internet of things. Among these, mobile health (mHealth) apps are one type of strategy that allows us to perform tasks such as modernizing data acquisition analysis for clinical trials, facilitating behavior change among users, disease management, self-diagnosis, improving patients’ confidence and satisfaction, and reducing health care costs [1-4].

Several studies have examined the use of mHealth for environmental health impact assessments—assessment of health-related problems deriving from the environment, such as chemical hazards, environmental contaminants, and other aspects of the ambient and living environment [5]. For example, Karagiannaki et al [6] deployed mHealth to monitor environmental factors affecting maternal health remotely. Honkoop et al [7] predicted the onset of asthma according to physiological, behavioral, and environmental data obtained by mHealth and home-monitoring sensors. Another study emphasized the importance of integrating location-based services into mHealth platforms to evaluate exposure to air pollutants [8]. As these examples show, mHealth is being actively used to promote human health in the context of environmental risk.

Using mHealth alongside medical prescriptions for older adults is more effective than traditional methods of managing their health [9]. mHealth positively influences health behavior, including improving physical activity, normalizing BMI, and decreasing sedentariness [10-12]. Although older people know the benefits of improving health behaviors, they tend not to pursue such improvements. In such cases, mHealth can facilitate older people’s healthy behavior through notifications that serve as reminders [13,14]. Furthermore, older people need to extend their health span through continuous and systematic health management [15]; managing chronic diseases is the most important and fundamental aspect in this regard. mHealth is also effective for people with chronic diseases [16,17]. One study proved that mHealth was useful in meeting older people’s information needs, especially concerning health, and was entirely accepted as a tool for monitoring health status and changing behavior [18]. Clearly, the evidence suggests that mHealth is an effective means of health management for older people and that the efforts should be focused on increasing its usage. Thus, studying the acceptability of mHealth usage among older people is important. In this study, we examine the acceptability of a personalized health care service app that we are currently developing.

Research on technology acceptance commonly uses the technology acceptance model (TAM) and the unified theory of acceptance and use of technology (UTAUT). TAM [19] is widely used in research that primarily addresses the intention to use information and communications technology; it assesses a person’s attitude toward using the system, its perceived usefulness (PU), and its perceived ease of use (PEOU). Attitude directly affects behavioral intention to use the system, and PU and PEOU indirectly affect behavioral intention by directly affecting attitude. UTAUT [20] is a model developed by analyzing and comparing 8 models related to behavioral intention; in this model, performance expectancy, effort expectancy, and social influence affect behavioral intention, whereas behavioral intention and facilitating conditions affect use behavior. Unlike TAM, the UTAUT does not include “attitude” in the model.

### Personalized Health Care Service App

The personalized health care service app we are developing aims to facilitate early identification and management of the health effects of exposure to real-time environmental risk factors. It provides health status reports and hospital visit recommendations based on the user’s biosignals and surrounding environment information. Biosignals such as electrocardiograms and heart rates are measured by wearable devices and linked with the app. Our app also assesses concentrations of hazardous substances in the air as environmental risk factors. It contains the values for particular matter (PM_2.5_, PM_10_), ozone, nitrogen dioxide, carbon monoxide, and sulfur dioxide. Instead of individually measuring the concentration of environmental risk factors, our design measures concentration data every minute using real-time personal location information based on the global positioning system combined with information from environmental harmful factor concentration stations in Korea. The app also provides information about health risks by considering individual health status (sociodemographic variables, individual medical checkups, diagnostic records, etc) and identifying correlations between environmental exposure and health effects. Furthermore, the app provides regular health analysis reports, information about nearby hospitals, recommendations for appropriate actions in the event of a health hazard, and so forth.

### Objective

This study aims to identify predictors of older people’s intention to use the personalized health care service app and to verify whether chronic disease moderates the effect on behavioral intentions using the extended UTAUT model.

## Methods

### Research Model and Research Hypotheses

We used the latent variables of performance expectancy, effort expectancy, social influence, and facilitating conditions from UTAUT [20]. UTAUT addresses actual use behavior; thus, facilitating conditions are set as variables that affect use behavior but not behavioral intention. However, since the app in this study is in the development stage and has not been released, use behavior cannot yet be measured. In the later UTAUT2 model developed by Venkatesh et al [21], facilitating conditions are extended to include factors affecting behavioral intention and use behavior. To receive customized services through the app, it is essential to use a wearable device to measure biosignals. Therefore, we also included device trust in our research model.

Performance expectancy is defined as “the degree to which individuals believe that using the personalized health management service app will help improve their health” [20]; this construct is similar to PU in the TAM [19]. Many previous studies on accepting health care services have demonstrated that performance expectancy is a good predictor of behavioral intention [22-35]. Thus, we proposed the following hypothesis.

H1: Performance expectancy will have a significant positive influence on behavioral intention.

Effort expectancy is also an essential predictor of behavioral intention; it refers to “the degree of ease associated with the use of the personalized health management service app” [20] and is similar to PEOU in the TAM [19]. The relationship between effort expectancy and behavioral intention has been confirmed in many studies [24-33,36]. Although the UTAUT model differs from TAM, assuming that effort expectancy does not affect performance expectancy, we can intuitively say that user-friendly and accessible services feel more useful. Indeed, consistent evidence shows a significant relationship between effort expectancy and performance expectancy [23-26,34,37,38]. Accordingly, we hypothesized the following.

H2: Effort expectancy will have a significant positive influence on behavioral intention.

H3: Effort expectancy will have a significant positive influence on performance expectancy.

Facilitating conditions are defined as “the degree to which an individual believes that an organizational and technical infrastructure exists to support the use of the personalized health management service app” [20]. In some cases, facilitating conditions are divided into resource and technological aspects [26]. In this study, we considered technological facilitating conditions at the organizational level. Particularly, older people unfamiliar with the new technology are more likely to try to use it if they have the support of a service provider. Previous studies have shown that facilitating conditions may affect behavioral intention [26-31,34]. Additionally, from the perspective of gerontechnology—which helps older people lead a consistently healthier, more independent, and more socially engaged life—a study predicted that facilitating conditions will affect performance expectancy [38]. From the health management perspective, some studies showed that facilitating conditions can influence performance expectancy [26,32,34,38]. Taking these points together, we proposed the following 2 hypotheses.

H4: Facilitating conditions will have a significant positive influence on behavioral intention.

H5: Facilitating conditions will have a significant positive influence on performance expectancy.

Social influence refers to “the degree to which an individual perceives that important others believe he or she should use the personalized health management service app” [20]. If people who are important to target users want them to use particular services, their usage likelihood increases. In particular, this influence may be more decisive when their knowledge about the service is insufficient or when the service is unfamiliar. Many studies have shown that social influence can be one of the good predictors of behavioral intention [25-29,32-34,37]. Accordingly, we hypothesized the following.

H6: Social influence will have a significant positive influence on behavioral intention.

Device trust is defined as “the degree to which individuals believe that they are confident in the quality and reliability of wearable devices” [39]. Although many studies have verified the accuracy and reliability of wearable devices from the perspective of precision medicine, there are relatively few studies on their technological acceptance [36]. Considering the technology behind wearable medical devices has reached a certain level of maturity, we need to pay attention to other aspects. In particular, if users can trust the measurement function and security of wearable devices, their intention to use the health care service will increase. Several studies have shown that trust in products or services has a positive effect on behavioral intention toward new technology [24,27,37]. Our app will provide personalized services based on biosignals measured by wearable devices. Therefore, those who trust wearable devices should be more likely to evaluate this service model as useful [40]. Several similar studies have found positive relationships between trust and performance expectancy [25,37,41]. Accordingly, we hypothesized the following:

H7: Device trust will have a significant positive influence on performance expectancy.

H8: Device trust will have a significant positive influence on behavioral intention.

It is widely known that people with chronic diseases are more vulnerable to environmental risk factors such as air pollution [42,43]. Furthermore, patients with chronic disease are more likely to use health system portals and to track health indicators [44]. Therefore, we assumed that patients with chronic disease would show more interest in using health care services for health management than other patients. Moreover, since patients with chronic disease periodically measure and manage their biosignals, such as blood pressure and blood sugar, they would be more accustomed to biosignal measurement. Accordingly, we hypothesized the following:

H9-H11: The influences of performance expectancy on behavioral intention (H9), device trust on behavioral intention (H10), and device trust on performance expectancy (H11) are moderated by the presence or absence of chronic diseases, such that the influences will be stronger for people who have chronic diseases.

**Figure 1:**
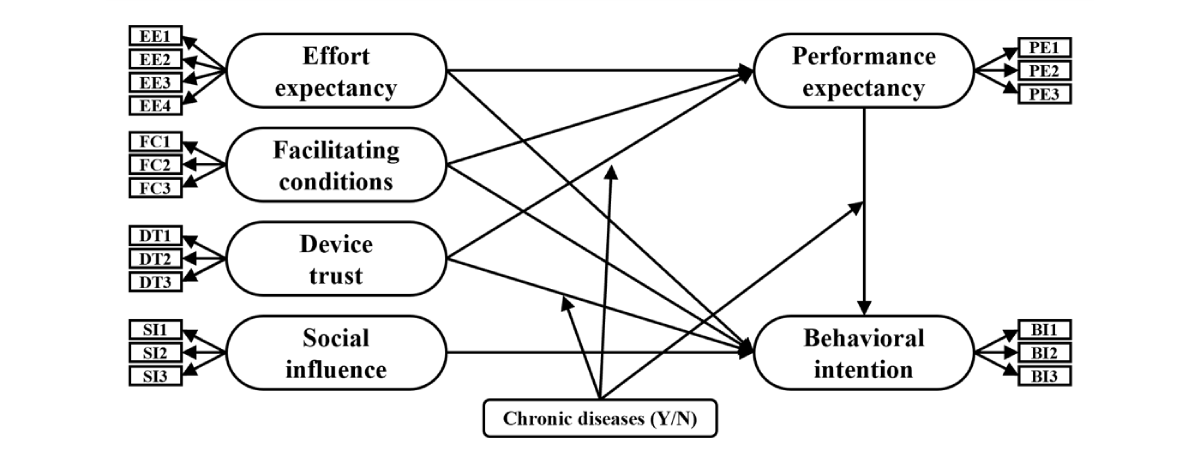
Research model. BI: behavioral intention; DT: device trust; EE: effort expectancy; FC: facilitating conditions; PE: performance expectancy; SI: social influence.

In summary, the proposed research model is shown in Figure 1.

### Data Collection

The web-based survey was conducted from June 27 to July 4, 2022, by the survey company dataSpring. The sample size was calculated using an a priori sample size calculator for structural equation modeling (SEM) [45]. The minimum recommended sample size was 200 cases based on 6 latent and 19 observed variables, with an anticipated effect size of 0.3, a desired statistical power level of 0.9, and a probability level of .05.

The study’s target group comprised people aged between 60 and 75 years and who are vulnerable to environmental risk factors. We recruited a sample of 500 participants to perform a multigroup analysis. The survey was first conducted on 290 people with chronic diseases, recruited using convenience sampling; subsequently, 210 people without chronic diseases were surveyed by matching the intergroup gender and age ratios as much as possible.

Only individuals who read the description of the research before the survey and voluntarily agreed to participate were selected as study participants. Before answering the questionnaire, they watched a video explaining the functionality of the personalized health care service app, which took about 4 minutes.

### Ethics Approval

This study was conducted after receiving ethical approval from the institutional review board of Yonsei University Wonju Severance Christian Hospital (CR322027).

### Questionnaire Development

All questionnaire items were constructed based on previous studies (see Multimedia Appendix 1). The measurement variables used in the research model were scored on 5-point Likert scales ranging from 1=“strongly disagree” to 5=“strongly agree.” Additionally, information about gender, age, residential area, final educational background, and presence of chronic disease were collected. Residential areas were divided into metropolitan areas (Seoul, Incheon, and Gyeonggi) and others. A person with chronic disease was defined as “someone who has been diagnosed by a doctor and has been regularly under treatment or taking medication for at least three months”; participants were asked to self-report the presence of chronic diseases.

### Statistical Analysis

First, the general characteristics of the survey respondents were confirmed through frequency analysis. Subsequently, we verified the research model’s convergent and discriminant validity. Convergent validity was confirmed using factor loading, average variance extracted (AVE), and construct reliability values obtained through confirmatory factor analysis. Two methods were used to test the model’s discriminant validity. The first involved testing whether the construct’s square root value of AVE was greater than its correlation with any other constructs [46]. The second involved testing whether the range of adding or subtracting the SE of covariance multiplied by 2 to the correlation coefficient between 2 latent variables did not include 1 [47,48]. Additionally, to verify the cross-validation between the chronic disease and nonchronic disease groups, an analysis of measurement equivalence was conducted.

Then, SEM—a method used to statistically verify relationships defined in a theoretical framework using the covariance or correlation matrixes of the data—was performed to verify the research hypotheses. The analysis was performed using maximum likelihood estimation, and the model fit was confirmed through the absolute and incremental fit indexes. For the former, normed chi-square, goodness-of-fit index, and root-mean-square error of approximation were used. For the latter, Tucker-Lewis index, comparative fit index, and normed fit index were used. Subsequently, the critical ratio for differences was confirmed by restricting each pathway to verify the moderating effect of chronic diseases. Bootstrapping using the maximum likelihood method was repeated 10,000 times to confirm the statistical significance of the model’s indirect effects. Finally, statistical significance and confidence limits were obtained with the bias-corrected percentile method [49]. All statistical analyses were performed using SPSS Statistics 26.0 and SPSS Amos 28.0 Graphics (IBM Corp).

## Results

### General Respondent Characteristics

The descriptive statistics for respondents’ characteristics are shown in Table 1. Among 500 participants, the responses of 23 participants were discarded due to poor data quality, such as straight-lined answers. Given the methodological procedure described above (in which nonchronic disease participants were recruited after those with chronic diseases and were matched for gender and age), there were no statistically significant differences between the 2 groups according to gender or age. Additionally, we found no statistically significant differences between the groups in terms of residential area and educational background.

**Table 1:** Descriptive statistics for respondents’ characteristics.

Characteristics			Total	CD^a^		No CD	P value	
Total, n (%)			477 (100)	278 (58.3)		199 (41.7)	N/A^b^	
**Sex** **, n (%)**								.55
	Male	276 (57.9)		164 (59.4)	112 (40.6)			
	Female	201 (42.1)		114 (56.7)	87 (43)			
**Age (years)**								.63
	60-64, n (%)	280 (58.7)		159 (56.8)	121 (43.2)			
	65-69, n (%)	150 (31.4)		89 (59.3)	61 (41)			
	70-75, n (%)	47 (10)		30 (63.8)	17 (36)			
	Continuous mean (SD)	64.31 (3.42)		64.46 (3.47)	64.09 (3.36)		.23	
**Residential area,** **n (%)**								.92
	Metropolitan areas	308 (64.6)		180 (58.4)	128 (41.6)			
	Others	169 (35.4)		98 (58)	71 (42)			
**Educational background**, **n (%)**								.69
	High school or lower	152 (31.9)		91 (59.9)	61 (40)			
	College or university	274 (57.4)		160 (58.4)	114 (41.6)			
	Graduate school	51 (11)		27 (53)	24 (47)			
**Chronic diseases** **, n (%)**								N/A
	Yes	278 (58.3)		N/A	N/A			
	No	199 (41.7)		N/A	N/A			

^a^CD: chronic diseases.

^b^N/A: not applicable.

### Validity Analyses

#### Convergent Validity

The results of the confirmatory factor analysis are shown in Table 2. According to Hulland [50], the value of standardized factor loading is recommended to be 0.7 or higher. Bagozzi and Yi [51] suggested that the construct reliability and AVE values should be greater than or equal to 0.7 and 0.5, respectively. With these criteria, confirmatory factor analysis confirmed the convergent validity of our model.

**Table 2:** Confirmatory factor analysis.

Variables		β	Estimate	SE	Critical ratio	P value	AVE^a^		CR^b^	
**PE^c^**								0.74		0.89
	PE1	.70	1.00	—^d^	—	—	—		—	
	PE2	.82	1.30	0.08	16.19	<.001	—		—	
	PE3	.84	1.21	0.07	16.67	<.001				
**EE^e^**								0.68		0.89
	EE1	.69	1.00	—	—	—	—		—	
	EE2	.80	1.39	0.09	15.49	<.001	—		—	
	EE3	.79	1.41	0.09	15.39	<.001	—		—	
	EE4	.79	1.40	0.09	15.41	<.001	—		—	
**FC^f^**								0.68		0.86
	FC1	.70	1.00	—	—	—	—		—	
	FC2	.78	1.21	0.08	15.34	<.001	—		—	
	FC3	.80	1.27	0.08	15.70	<.001	—		—	
**SI^g^**								0.67		0.86
	SI1	.71	1.00	—	—	—				
	SI2	.75	1.15	0.08	15.16	<.001	—		—	
	SI3	.81	1.27	0.08	16.10	<.001				
**DT^h^**								0.62		0.83
	DT1	.69	1.00	—	—	—	—		—	
	DT2	.71	1.29	0.10	13.18	<.001	—		—	
	DT3	.79	1.35	0.10	14.16	<.001	—		—	
**BI^i^**								0.77		0.91
	BI1	.83	1.00	—	—	—	—		—	
	BI2	.83	1.09	0.06	19.56	<.001	—		—	
	BI3	.79	1.02	0.06	18.62	<.001	—		—	

^a^AVE: average variance extracted.

^b^CR: construct reliability.

^c^PE: performance expectancy.

^d^Not available.

^e^EE: effort expectancy.

^f^FC: facilitating conditions.

^g^SI: social influence.

^h^DT: device trust.

^i^BI: behavioral intention.

#### Discriminant Validity

The discriminant validity was tested in 2 ways. Table 3 shows the results of the first method, which compares the correlation coefficient of the latent variables and the square root of AVE. Although discriminant validity was generally satisfied, we found that some correlation coefficients were higher than the square root of AVE. Next, discriminant validity was reconfirmed by the second method, using performance expectancy and social influence, which had the highest correlation coefficients. The correlation coefficient between them was 0.866, and the SE of covariance was 0.024; therefore, we confirmed that the range of adding or subtracting the SE multiplied by 2 to the correlation coefficient did not include 1.

**Table 3:** Discriminant validity analysis.

	PE^a^	EE^b^	FC^c^	SI^d^	DT^e^	BI^f^
PE	1					
EE	0.75	1				
FC	0.87	0.76	1			
SI	0.87	0.71	0.85	1		
DT	0.73	0.64	0.76	0.78	1	
BI	0.72	0.53	0.65	0.77	0.65	1
Sqrt of AVE^g^	0.86	0.83	0.82	0.82	0.79	0.88

^a^PE: performance expectancy.

^b^EE: effort expectancy.

^c^FC: facilitating conditions.

^d^SI: social influence.

^e^DT: device trust.

^f^BI: behavioral intention.

^g^Sqrt of AVE: square root of average variance extract.

#### Cross-Validity

The verification of measurement equivalence was performed through multigroup confirmatory factor analysis. To confirm the difference between the unconstrained and factor-loading constrained models, we used the difference between their chi-square values. The chi-square value of the unconstrained model was 591.4, and the degree of freedom was 274; for the factor loading constrained model, the values were 603.9 and 287, respectively. Since the threshold of the chi-square value at the degree of freedom of 13 was 22.4, there was no statistically significant difference in factor loading between the 2 groups for the measurement tool.

### Hypotheses Testing

We performed SEM to test our research hypotheses. Table 4 shows that H1, H5, and H6 were supported. That is, participants who stated that performance expectancy and social influence were important were more likely to have behavioral intentions to use mHealth, and people who thought that facilitating conditions was important were more likely to believe performance expectancy was also important. Bootstrapping was performed 10,000 times to check whether there was an indirect effect, whereby facilitating conditions affected behavioral intention through performance expectancy. Assessing the statistical significance using the bias-corrected percentile method showed that the standardized indirect effect of facilitating conditions on behavioral intention was 0.325, the significance probability 0.006, and the 95% CI of 0.115-0.759, confirming a significant indirect effect.

The model fit of SEM was confirmed through normed chi-square, goodness-of-fit index, and root mean squared error of approximation (absolute fit index), Tucker-Lewis index, comparative fit index, and normed fit index (incremental fit index). Therefore, we confirmed that the recommended values suggested in previous studies were generally satisfied (Table 5).

The results of multigroup SEM analysis are shown in Table 6. The significance of the path difference between groups can be confirmed by looking at the critical ratio for differences. If the absolute value of the critical ratio is 1.965 or higher, there is a statistically significant difference in the path coefficients between groups. As the critical ratio for differences in hypothesis 11 was –2.165, there is a statistically significant difference between the 2 groups in the effect of device trust on performance expectancy. Whether there was an indirect effect of device trust on behavioral intention through performance expectancy in the chronic disease group was also confirmed through bootstrapping. Results found that the standardized indirect effect of device trust on behavioral intention was 0.122, the probability of significance 0.039, and 95% CI of 0.007-0.346, confirming that device trust has a significant indirect effect on behavioral intention in the chronic disease group.

**Table 4:** Verification of the research hypotheses.

Hypothesis	Path	β	Estimate	SE	P value
H1	PE^a^→BI^b^	.453	0.538	0.179	.003
H2	EE^c^→BI	–.097	–0.118	0.097	.23
H3	EE→PE	.116	0.120	0.070	.09
H4	FC^d^→BI	–.338	–0.385	0.265	.15
H5	FC→PE	.716	0.686	0.099	<.001
H6	SI^e^→BI	.693	0.776	0.197	<.001
H7	DT^f^→BI	.089	0.107	0.114	.35
H8	DT→PE	.122	0.123	0.074	.10

^a^PE: performance expectancy.

^b^BI: behavioral intention.

^c^EE: effort expectancy.

^d^FC: facilitating conditions.

^e^SI: social influence.

^f^DT: device trust.

**Table 5:** Model fit.

Model fit measure	Value	Recommended value	Results	Reference
Normed *χ*^2a^	3.4	≤3	Acceptable	Hair et al [52]
GFI^b^	0.9	>0.90	Acceptable	Hair et al [52]
RMSEA^c^	0.1	<0.08	Good	Hair et al [52]
TLI^d^	0.9	>0.90	Good	Bentler et al [53]
CFI^e^	0.9	>0.90	Good	Hair et al [52]
NFI^f^	0.9	>0.90	Good	Bentler et al [53]

^a^*χ*^2^_138_=463.3.

^b^GFI: goodness-of-fit index.

^c^RMSEA: root mean squared error of approximation.

^d^TLI: Tucker-Lewis index.

^e^CFI: comparative fit index.

^f^NFI: normed fit index.

**Table 6:** Verification of the research hypotheses on the moderating effect.

Hypothesis (path) and group			β		Estimate		SE		*P*-value		Critical ratio for differences	
**H9 (PE^a^→BI^b^)**												–0.934
	CD^c^	.438		0.516		0.188		.006				
	No CD	.429		0.508		0.393		.429				
**H10 (DT^d^→BI)**												0.780
	CD	.060		0.069		0.146		.060				
	No CD	.215		0.277		0.224		.215				
**H11 (DT→PE)**												–2.165
	CD	.278		0.272		0.083		.001				
	No CD	–.100		–0.107		0.154		.487				

^a^PE: performance expectancy.

^b^BI: behavioral intention.

^c^CD: chronic disease.

^d^DT: device trust.

## Discussion

### Principal Results

This study identified the predictors of acceptance of a personalized health care service app by conducting SEM on questionnaire survey data collected from older adults aged 60 to 75 years. Performance expectancy (β=.453; P=.003) and social influence (β=.693; P<.001) were identified as significant predictors. Furthermore, bootstrapping analysis confirmed that facilitating conditions (β=.325; P=.006; 95% CI 0.115-0.759) had an indirect effect on behavioral intention. Differences between groups according to the presence or absence of chronic diseases were confirmed through multigroup SEM. Additionally, device trust (β=.122; P=.039; 95% CI 0.007-0.346) was found to have a significant indirect effect on behavioral intention in patients with chronic disease.

### Comparison With Prior Work

As expected, performance expectancy was a significant predictor of the intention to use the personalized health care service app. These results were in line with many previous studies on the intention to use health care–related services [22-35]. Performance expectancy can be increased if it is possible to integrate mHealth services, with an existing health tracking app or a health information app that includes medication, treatment, and health checkup histories or hospital information such as the nearest hospital or reservation service. Park et al [29] emphasized the importance of effectively expressing the causal relationship between personal health records and physiological conditions and providing immediate feedback from health experts for encouragement to use a health care app. Another study suggested that mHealth apps should be integrated with other applications [28]. Notably, the results of a previous study indicate that performance expectancy has an important effect on the behavioral intention of people who have never used the service [54].

In line with several studies [22,23,35,37,41], we could not find evidence for effort expectancy’s effect on behavioral intention. The lack of direct effect might be because our survey design targeted people in the preuse stage [23,35]. Another study that found no effect of effort expectancy suggested the following explanation: their study sample was already familiar with the service, so variance according to effort expectancy was minimal [37]. In this study, although the service was explained through a description of the research and an introductory video, it may have been difficult for respondents to judge how much effort would actually be required.

Facilitating conditions were not a predictor of behavioral intention but did affect performance expectancy. We confirmed that facilitating conditions have an indirect effect on behavioral intention through performance expectancy. Some studies have revealed that facilitating conditions have an indirect effect on behavioral intention through performance expectancy [26,32,34]. In other words, if there is support from the service provider, the user may feel that the service is more useful. Since the participants in this study are older people who are unfamiliar with new technology, the facilitative infrastructure of service providers is important. One study showed that performance expectancy was relatively more important than facilitating conditions in a group that had not yet experienced the service [54]. However, in the case of the group with experience in using the service, the importance of facilitating conditions was relatively high. With the previous study’s results in mind, it would be interesting to investigate any changes in our results after our mHealth app has been put into actual use.

Social influence was also an essential predictor of the intention to use the service model; this supports previous studies dealing with similar topics to ours, which have reported that social influence positively affects behavioral intention [22,30,31,35]. There are several possible explanations, though 1 important reason could be the cultural context. Older people’s decisions regarding health care may be more influenced by their families than by themselves; the influence of the family is particularly prominent in Confucian culture [55]. For this reason, the personalized health care service app will need to be promoted at the family and community levels in such cultures especially.

Finally, we confirmed that trust in wearable devices affects performance expectancy in the group with chronic diseases. Through this, we proved that device trust could be one of the predictors that indirectly affected behavioral intention. Artificial intelligence–based health care management services using biosignal measurement and wearable devices are known to be safe and cost-effective for managing chronic diseases [56]. Thus, in the early stage of service introduction, the project should specifically target older people with chronic diseases vulnerable to environmental risk factors. Improving their performance expectancy would be helpful, highlighting the accuracy and reliability of wearable devices.

### Limitations

In this study, discriminant validity was verified in 2 ways. There were some ambiguities in comparison with the correlation coefficient between latent variables and the square root of AVE. These can be solved by merging the corresponding latent variables or removing some [57]; however, this method is not desirable when the research model is built based on a particular theory [48]. Since UTAUT is a very widely used theory, we decided to accept the conclusion gained using the second method of testing discriminant validity used in this study. In future studies, better results could be obtained to reduce measurement errors.

The mHealth app, which we are developing, does not target a specific chronic disease. Therefore, in this study, the definition of “chronic disease” has been set broadly. There are various types of chronic diseases, and the methods and levels of their management are also different. More detailed research can be done on mHealth for the management of specific chronic diseases.

Additionally, since this study was designed as a cross-sectional survey and random sampling was not applied, the generalizability of the results is limited. The personalized health care service app introduced in this study is currently under development; the goal is to develop it into a more user-friendly service through continuous research on the attitudes of potential users. Furthermore, continuous research, which can better represent the population, will further strengthen the explanatory power of the model proposed in this study.

### Conclusions

Performance expectancy, social influence, and facilitating conditions are predictors of the intention to use mHealth among older people vulnerable to environmental risk factors. It is important to demonstrate and highlight the benefits of personalized health care services for health management to encourage older people to use them. The awareness of people around the target users also plays an important role. In particular, it is necessary to promote such services at the family and community levels; this aspect is critical in the Confucian culture. In addition, support from service providers should be strengthened so that older people can trust that they have consistent access to technical support. Furthermore, our findings suggest that different strategies should be used depending on the presence or absence of chronic disease. The reliability of biosignal measurements made by wearable devices should be emphasized to achieve a higher usage rate among older people with chronic diseases.

## Appendix

Multimedia Appendix 1 Overview of the instrument.

## Abbreviations

AVE: average variance extracted
mHealth: mobile health
PEOU: perceived ease of use
PU: perceived usefulness
SEM: structural equation modeling
TAM: technology acceptance model
UTAUT: unified theory of acceptance and use of technology
