# Supplementary material for: Factors Predicting Older People’s Acceptance of a Personalized Health Care Service App and the Effect of Chronic Disease: Cross-Sectional Questionnaire Study
Source: JMIR Aging. 2023 Jun 21;6:e41429. doi: 10.2196/41429 (PMC10334719; doi:10.2196/41429)
Supplement: Multimedia Appendix 1 [file aging_v6i1e41429_app1.docx]

Multimedia Appendix 1. Overview of the instrument.

| Latent variable | Items | Reference |
| --- | --- | --- |
| Performance expectancy | - The personalized healthcare service model for the management of exposure to environmental risk factors would be useful in managing my health. - Using the personalized healthcare service model for the management of exposure to environmental risk factors would be able to manage my health more quickly. - Using the personalized healthcare service model for the management of exposure to environmental risk factors would be helpful in managing my health. | [20,29] |
| Effort expectancy | - Learning how to use the personalized healthcare service model for the management of exposure to environmental risk factors would be easy for me. - My interaction with the personalized healthcare service model for the management of exposure to environmental risk factors would be clear and understandable. - I find the personalized healthcare service model for the management of exposure to environmental risk factors easy to use. - It would be easy for me to become skillful at using the personalized healthcare service model for the management of exposure to environmental risk factors. | [21] |
| Social influence | - My family members think I should use the personalized healthcare service model for the management of exposure to environmental risk factors. - People around me would support my use of the personalized healthcare service model for the management of exposure to environmental risk factors. - People who give me advice would support my use of the personalized healthcare service model for the management of exposure to environmental risk factors. | [21] |
| Facilitating conditions | - I believe the guidance will be available to me when deciding whether to use the personalized healthcare service model for the management of exposure to environmental risk factors. - I believe specialized instructions concerning the use of the personalized healthcare service model for the management of exposure to environmental risk factors will be available to me. - I believe I can be able to get help from the service provider when I have difficulties in using the personalized healthcare service model for the management of exposure to environmental risk factors. | [21,31] |
| Device trust | - I believe that wearable devices for measuring bio-signals are reliable. - I believe that my personal information will be safely protected when measuring bio-signals through wearable devices. - I believe that wearable devices for measuring bio-signals will provide accurate information. | [39] |
| Behavioral intention | - I am willing to use the personalized healthcare service model for the management of exposure to environmental risk factors in the future. - If the service is introduced, I would likely use the personalized healthcare service model for the management of exposure to environmental risk factors in my daily life. - If the service is introduced, I predict that I would use the personalized healthcare service model for the management of exposure to environmental risk factors. | [20] |

1=strongly disagree, 2=disagree, 3=neutral, 4=agree, 5=strongly agree.
